# Stereomicroscopic and Microscopic Study of Dental Structural Aspects Derived from Iatrogenic and Pathological Processes Suffered by a Second Mandibular Premolar - A Case Study

**DOI:** 10.25122/jml-2020-0181

**Published:** 2020

**Authors:** Ioana Suciu, Mihaela Chirila, Mihai Ciocardel, Ioana Ruxandra Bartok, Oana Amza, Bogdan Dimitriu, Monica Voiculeanu, Magdalena Anca Coricovac

**Affiliations:** 1. Department of Endodontics, Faculty of Dental Medicine, “Carol Davila” University of Medicine and Pharmacy, Bucharest, Romania; 2. Department of Petroleum Geology and Reservoir Engineering, Petroleum-Gas University of Ploiesti, Romania; 3. Department of Esthetics, Faculty of Dental Medicine, “Carol Davila” University of Medicine and Pharmacy, Bucharest, Romania; 4. Discipline of Embryology, Faculty of Dental Medicine, “Carol Davila” University of Medicine and Pharmacy, Bucharest, Romania

**Keywords:** Stereomicroscopy, microscopy, warm lateral condensation technique

## Abstract

Microscopic studies performed on extracted human teeth after their preparation in advance is helpful in a relatively good reestablishment of the treatment steps that have been applied to these teeth, as well as an evaluation of the quality of such treatments. Therefore, we have used stereo- and optical microscopy, highlighting aspects of external morphology, as well as root canal space of an extracted mandibular second premolar, subjected to prosthetic and endodontic treatment. In order to verify some technical errors that might occur during the endodontic and restorative treatment, we tried to appreciate the quality of the root canal filling and cervical defect and access cavity restoration of an extracted premolar #45.Without having the data from clinical records, we concluded that the method used for root canal filling was the warm lateral condensation technic; we also appreciated the quality of the fusion of the gutta-percha cones used, so the introduction of heated spreaders only in the central part of the bunch of cones makes it possible to clearly detect the boundaries between these cones towards the outside of the filling.

## Introduction

Ideally, microscopy studies should be correlated with data on the stages of treatment in the patient’s medical record in order to have a clear picture of therapeutic events. The information from the clinical records would also provide valuable knowledge of the chronology of events and would allow those who do the microscopic study to make some assessments regarding the evolution of the endodontic pathology. Obviously, the researchers who perform the post-extraction microscopic study notice, on the one hand, changes in the normal structure of the tooth as an expression of the pathological processes carried out and, on the other hand, changes caused by endodontic and restorative treatments [1,4].

## Material and Methods

We consider that a complete optical microscopic study firstly involves the use of a stereomicroscope and then an optical microscope. Therefore, microscopic investigations are performed in two stages: one stage before and one after sectioning the tooth with a microtome. In the first stage, aspects of external morphology are highlighted - stereomicroscopy is more useful here. In the second stage, the endodontic space and the relations between the different tissue components and the restorative ones or those used for endodontic treatments are explored microscopically. In this second stage, we applied both stereomicroscopy and optical microscopy in transmitted light, which allows higher degrees of magnification (over 20-25x). For the application of light microscopy in transmitted light, the dental fragments obtained after the first sectioning procedures are usually resected in order to obtain slices with thicknesses between 300-700 mm. We used these thin slices for the last stages of the study, the so-called thin sections, either as definitive or temporary microscopic preparations.

The stereomicroscopic study was performed both before and after the tooth sectioning with a stereomicroscope with a built-in professional digital camera (Leica EZ4D). The sections were made with a microtome (Smart Cut 6010 OKAM Ind. SuperhardTools, Valencia, CA, USA).

For the stereomicroscopic optical study, reflected light and magnification orders were mainly used, depending on the nature of the aspects that were captured.

A Leica DM LP microscope was used for the optical study in polarized light (various working variants). For this purpose, details that cannot be captured with the stereomicroscope could be detected. To use this microscope, the initial fragments were resected with the microtome into slices with a thickness of about 0.7-0.8 mm.

As a tool for performing microscopic study in light transmitted on thin sections, we used polarizing microscopes without necessarily using their analyzing polarizing optical filters. These microscopes are usually designed for research and offer greater versatility in use for detecting aspects of interest. However, where necessary, the use of analyzing polarizing filters allows the detection of aspects that cannot be highlighted by classical microscopy under certain conditions. This is due to the special properties of the birefringence of the investigated structures. These microscopes also allow the performance of precision micrometric measurements with a maximum possible error of ± 2 mm.

## Case report

A 56-year-old patient presented to the University Clinic of Endodontics, Faculty Of Dental Medicine, Bucharest, with tooth #45 affected by significant mobility (third-degree). The tooth had to be extracted at the time of the patient’s presentation, and informed consent was obtained to perform the present study. The patient was told it would be a microscopic study. However, the patient did not allow us to take photos of the oral cavity or the partial mandibular prosthesis, which was anchored with a very rigid hook on this tooth.

The patient reported that the partial acrylic denture had been used for more than three years. According to her report, this mobilizable prosthesis was relatively comfortable only in the first 3-4 months.

In the first phase, the patient noticed that this hook caused a lesion with a lack of substance in the cervical region due to the fact that the partial prosthesis did not have good stability.

The patient presented to another clinician than the one who made the partial denture, who tried to restore the tooth by repeatedly covering the cervical defect with restorative materials. He also recommended making another partial denture and another anchorage system on the remaining teeth. The patient refused, being at the moment satisfied with the restoration of the cervical defect. The same clinician showed the patient that the tooth shows an abrasion of the occlusal surface because of tooth #14, which is covered by an old prosthetic treatment with a metal ceramic crown.

Next, during the wearing of the partial acrylic prosthesis, the patient observes how the restorative material placed on the cervical region was largely dislocated by the wire hook in the functional acts that were done with the mobilizable mandibular prosthesis on the prosthetic field.

Subsequently, tooth #45 acquires increasing mobility and pulpal symptomatology, and the patient requested treatment from a third clinician, who also recommended changing the type of mobilizable prosthesis and leaving this tooth at rest. After pulpectomy of tooth #45, the clinician performed the root canal filling (using the hot lateral condensation technique) with coronary restoration.

After more than three years, the tooth, although painless, acquired accentuated mobility was described when the patient came to our Clinic. Given that, the poor stability of the mandibular acrylic partial denture was revealed.

The preparation of the teeth for the study supposes, in the opinion and practice of the authors, first of all, the removal of the organic residues (the adherent parts of the periodontal membrane) and the disinfection of the teeth for a sufficient period of time. However, it is not necessary to intervene in these biological samples by applying aggressive mechanical or chemical procedures in order not to change the structural aspects in these teeth and which we want to study in detail.

We appreciate that the successive immersion in several types of milder disinfectants for a period of time (2 weeks with NaOCl 3.5%, followed by irrigation with EDTA 10% solution and a final rinse with distilled water) ensures optimal cleaning [2]. Then, while working with these teeth, team members wear protective equipment equivalent to that worn during clinical practice with patients. Some specialists recommend autoclaving of the teeth that are subjected to research, ensuring thus “a microbiological safety” during the process. However, we do not agree with such practices because the temperatures during the autoclaving process are too high and not specific to natural biological conditions.

## Results and Discussions

**Figure 1:**
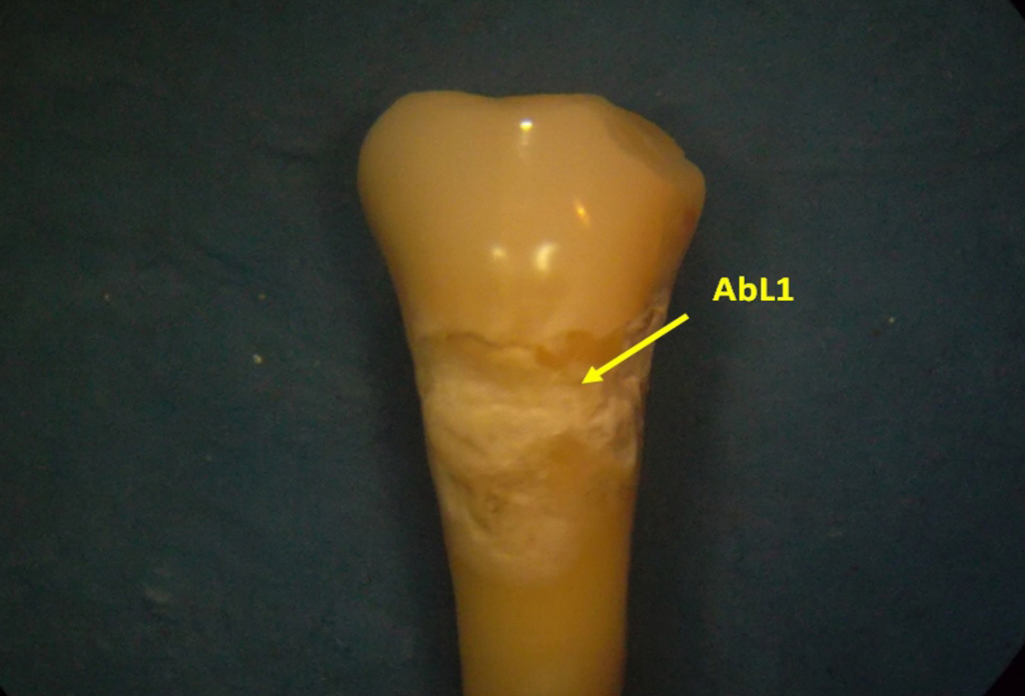
Vestibular image of the 4.5 premolar crown. In the cervical region, there is a wear lesion (abrasion lesion - AbL1) caused by a hook of a partial mandibular acrylic prosthesis worn by the patient over 3 years. By wearing the prosthesis, the hook completely mobilized the tooth (Stereomicroscopy, RL 8x). RL - reflected light; 8x - magnification order (eight, in this case).

**Figure 2:**
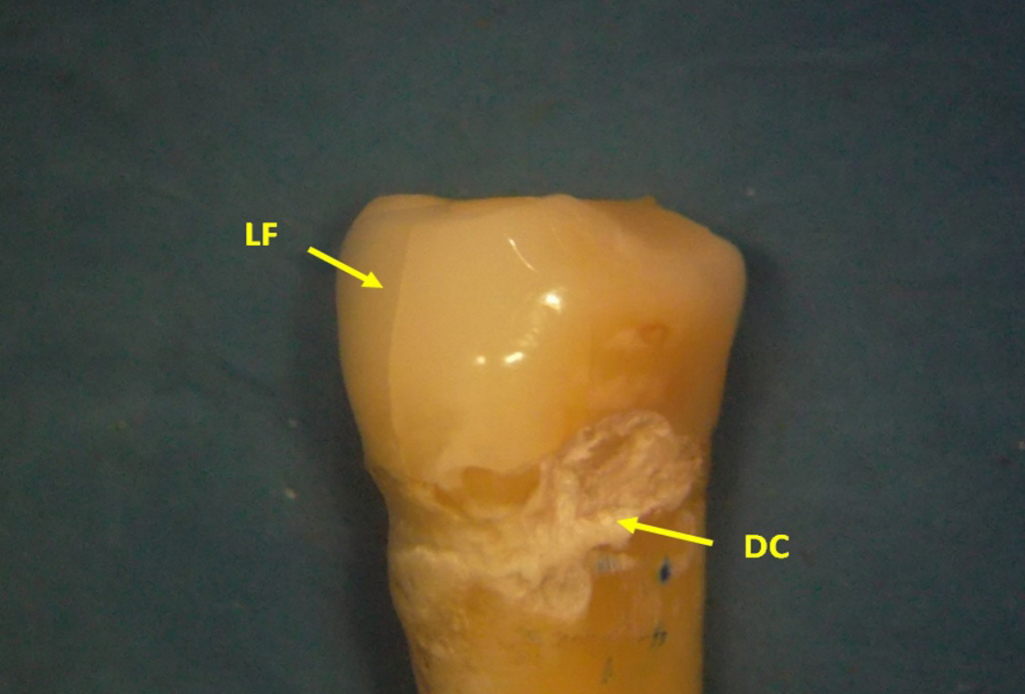
The premolar crown. In the middle of the buccal aspect, there is a longitudinal fissure (LF) that goes from the cervical to the occlusal surface (Stereomicroscopy, RL 8x). DC - dental cement.

**Figure 3:**
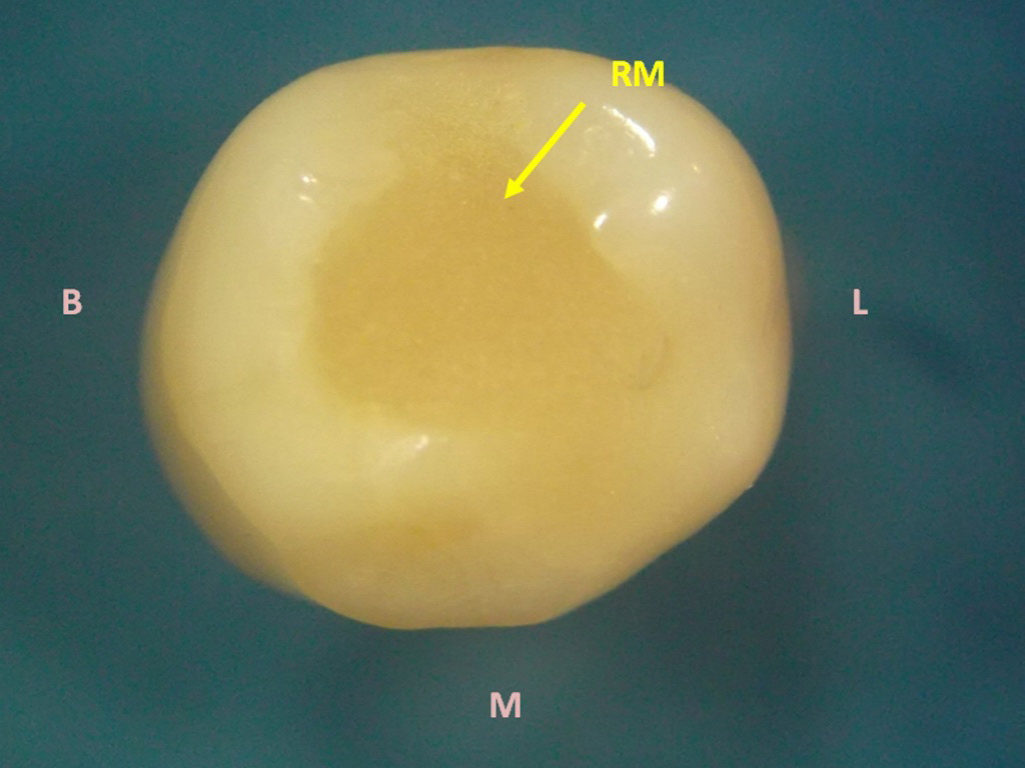
Occlusal image of the premolar crown. A restoration of the access cavity with restorative material (RM) was performed after pulpectomy of tooth #45. B - buccal; M - mesial, L - lingual.

**Figure 4:**
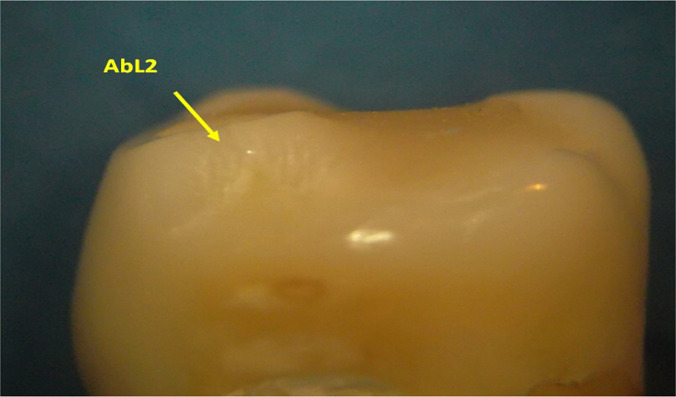
The mesial aspect of the premolar crown. In the left half of the occlusal surface of the crown, an abrasion facet (AbL2 - abrasion lesion) and small grooves with approximately vertical direction are 2) are also observed.

There is a special, less known method of creating the access tunnel to the pulp chamber, as follows: the coronal half is flared towards the occlusal, and its apical half is flared towards the pulp chamber [1, 3]. This approach was considered in the past (the ’90s), when the adhesion of composite materials was not very good, as an assurance that the fillings will not be dislocated in respect with the coronary region, nor will clog towards the pulp chamber (Figures 1-4). This technique is currently unknown to many clinicians and has been applied in the past by a few.

In our case, it is unlikely that the doctor who made the access cavity tried such a practice because the preparation of the access tunnel is way too asymmetrical [1].

While wearing the prosthesis, a clinician attempted to "repair" the lack of cervical substance with restorative material that was, in turn, dislocated and abraded by the hook. Remains of a cement used are seen at the bottom of the defect (DC - dental cement) (Stereomicr, RL, 8x)

Pulpectomy was necessary, because after wearing the partial acrylic prosthesis, about 8-9 months, tooth #45 became symptomatic with a noticeable third-degree mobility (Stereomicr, RL, 12.5x).

This lesion is due to the contact with an antagonist tooth (14) with a metal ceramic crown, which transmitted the paraaxial occlusion force on the premolar #45, and contributed to the emergence of pathological mobility (Stereomicr, RL, 16x).

Also, there was another approach error, namely that the walls of the endodontic access cavity do not converge to the apical as natural [1], but rather have an "hourglass" arrangement. The maximum narrowing area of the access cavity is marked with "md" (minimum diameter).

This filling was performed using the hot lateral condensation of the gutta-percha technique. The almost perfect fusion of the gutta-percha cones is remarkable due to the heat applied.

**Figure 5:**
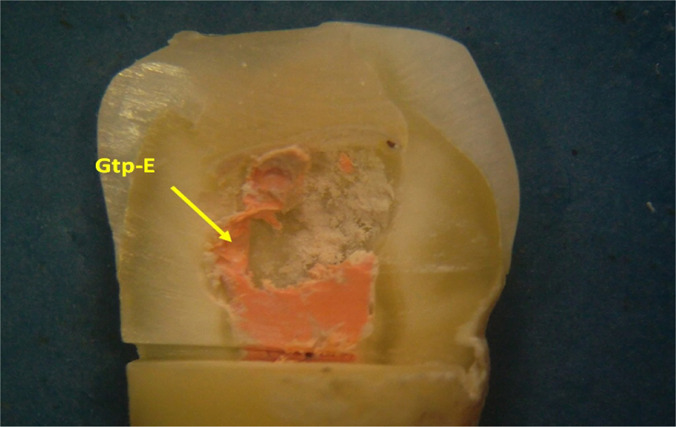
Appearance of the coronary pulp cavity after longitudinal sectioning of the crown of the tooth with a microtome in the vestibulo-oral direction. Findings: the pulp chamber is largely free, occupied by extensions of the gutta-percha cones (Gtp-E - gutta-percha extensions) remaining after sectioning with a heated instrument (Stereomicr, RL, 10x).

**Figure 6:**
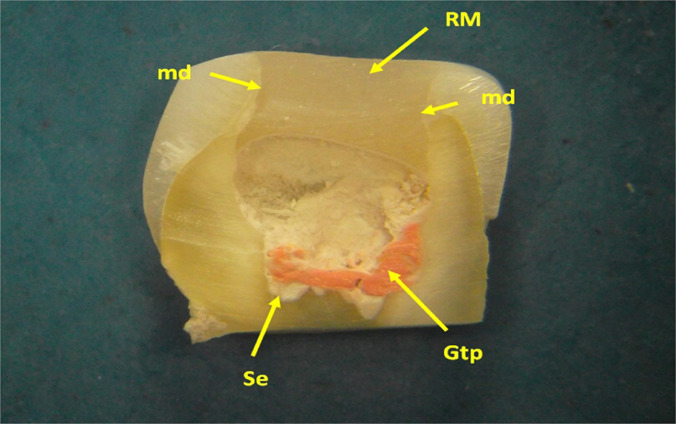
The other half of the crown was obtained by longitudinal sectioning. It is observed that the material that closes the coronary access cavity does not rest on any underlying material. Thus, the maintenance of the filling on the spot was strictly based on the adhesion to the buccal, lingual, mesial and distal walls of the access cavity. Remains of gutta-percha (Gtp) and endodontic sealer (Se) are observed at the base of the pulp chamber. RM - restorative material (Stereomicr, RL, 10x).

**Figure 7:**
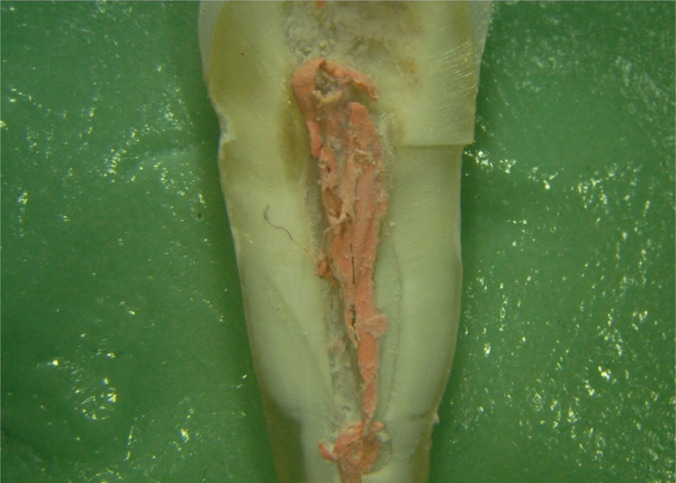
Upon longitudinal root sectioning, the root canal obturation detached from the walls of the premolar root canal (Stereomicr, RL, 8x).

**Figure 8:**
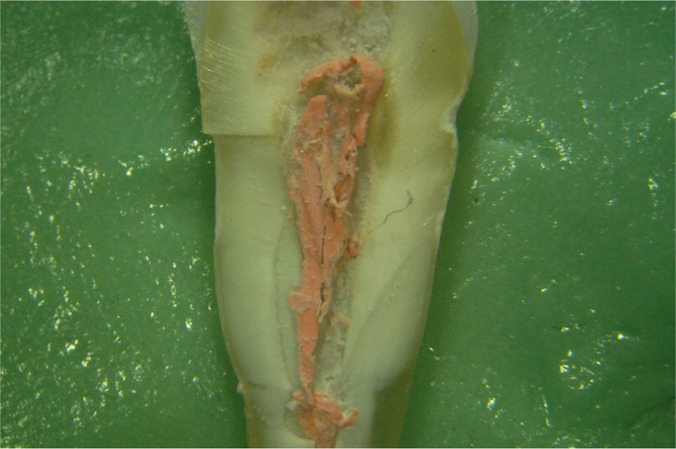
The canal obturation detached from the walls of the root canal of the premolar: its surface from the wall of the root canal (the canal obturation was turned by 180° and superimposed on the slope of the corresponding root half). The limits (LGP - limit between the gutta-percha points) between the master cone and the accessory cones are relatively well distinguished. Such a clear limit is marked by the arrows used for marking (arrows superimposed on the image). Portions with endodontic sealer are also seen in the picture (Se - sealer) (Stereomicr, RL, 8x).

Probably, this aspect with the boundaries between the gutta-percha cones that are more clearly visible on the external surface of the filling is due to the fact that the heated spreaders were always inserted centrally in the canal, between the gutta-percha cones already inserted [5, 6]. Thus, gutta-percha fluidized in the more central areas of the root canal and less towards its periphery (Figures 5-8).

**Figure 9:**
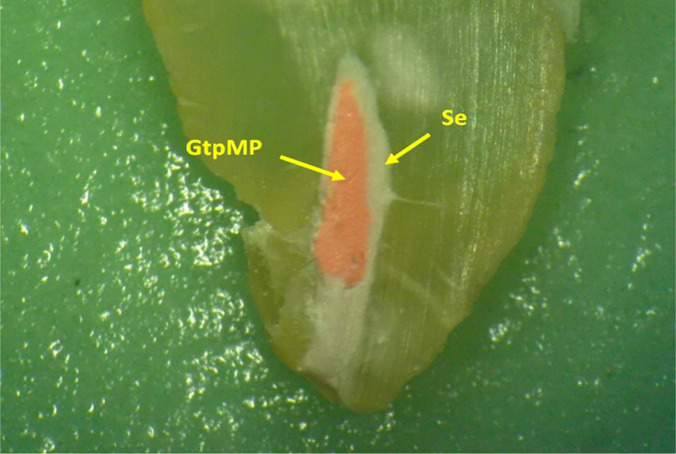
Detail on the apical portion, of one of the root halves obtained by longitudinal sectioning (Stereomicr, RL, 25x).

Although most of the canal obturation has come off, in the apical portion, photographed on the section plane is observed in the original position, the tip of the master cone (GtpMP - gutta-pecha master point), embedded in the sealer (Se) which that closes very well the space between the walls, root canal and cone (Figure 9). An apical constriction is not obvious, but canal obturation in this apical region can be considered quite successful, although the last millimeter of the canal contains only endodontic sealer.

This aspect of the apical region is given by a sudden change of direction of the root canal to the distal.

A possible explanation would be due to the fact that the restorative material is not a composite resin removed from the syringe, but a cement-like material, and resins, obtained by spatulation, spatulating was done less rigorously, air bubbles were embedded.

**Figure 10:**
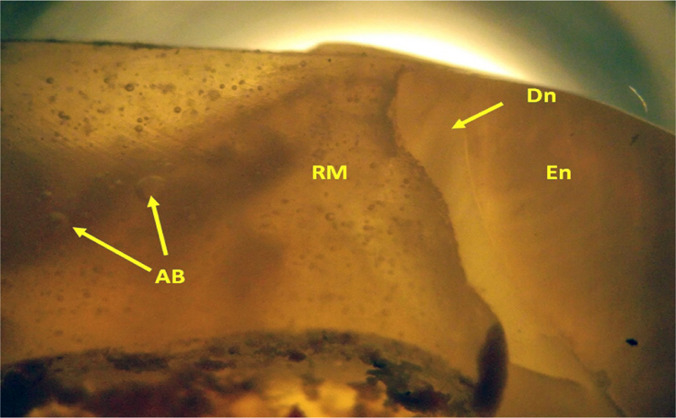
Detail image of the relationship of the coronary filling material with the rest of the remaining dental tissue in the occlusal area from a buccal aspect. We can observe the small amount of remaining dentin (Dn - dentin), tooth enamel (En - enamel) and the restorative material (RM) used which apparently, is full of air bubbles (AB - air bubbles). (LTP microscopy, 30x FFA).

**Figure 11:**
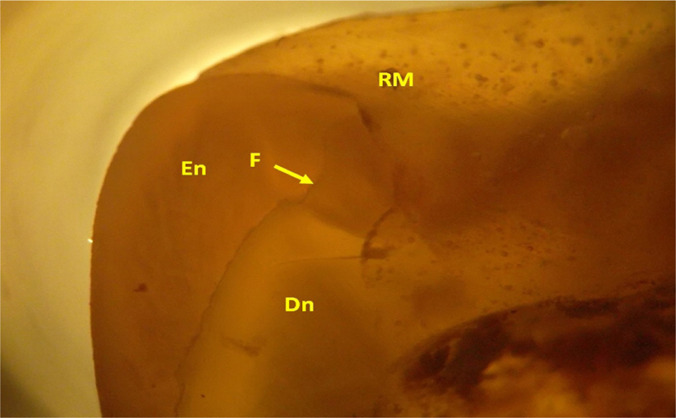
Detail image of the relationship of the coronary filling material with the rest of the remaining dental tissue in the occlusal area, from a lingual aspect. (LTP microscopy, 30x FFA). PTL - polarized transmitted light.

It is observed how the restorative material (RM) covered most of what was left of the lingual cusp (Figures 10, 11).

There is also a crack (F) in the remaining tooth enamel (En); this can be a factor that can lead to failure of the coronal restauration. Coronary dentin (Dn - dentin) can also be seen under the endangered cusp (enamel fissure is not propagated in the dentin).

**Figure 12:**
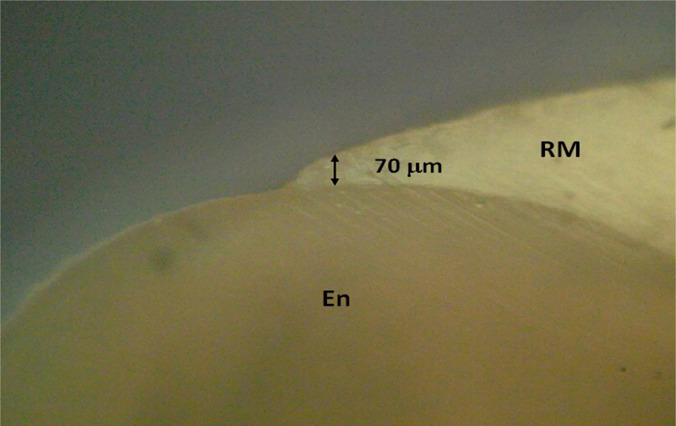
Detail: Restorative material (RM) - dental enamel (En - enamel) in the area of the lingual cusp. LTP microscopy, 40x CFA); PTL - polarized transmitted light; CFA - optical polarization analyzer with filter.

Using the micrometry technique, the thickness of the restoration material in the terminal part was established when passing on the enamel (about 70 μm) (Figure 12).

**Figure 13:**
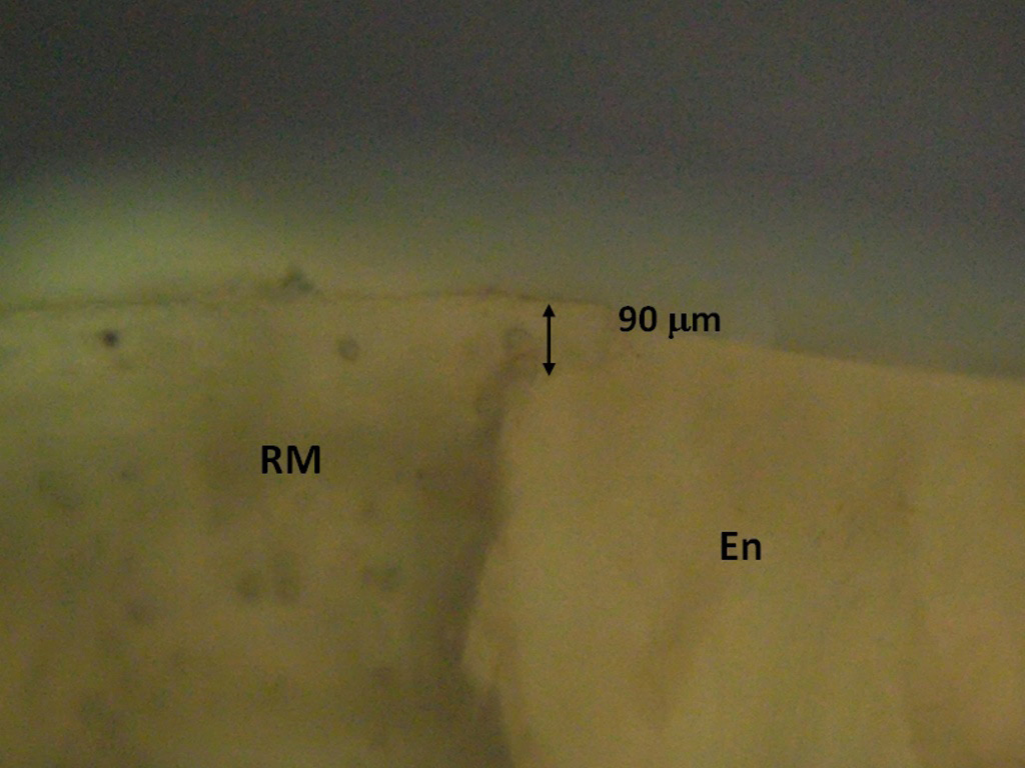
Detail: Junction between (restorative material (RM) and tooth enamel (En - enamel) in the area of the vestibular cusp. Here, the tracing between the two is clearer, although there is a thin edge of restorative material about the same thickness that was found in the area of the lingual cusp, specified above. (LTP microscopy, 40x CFA). PTL - polarized transmitted light; CFA-with optical analyzer polarizing filter.

Using the micrometry technique, the thickness of the restorative material in the terminal part was established when passing on the enamel (this is about 90 μm) (Figure 13).

**Figure 14:**
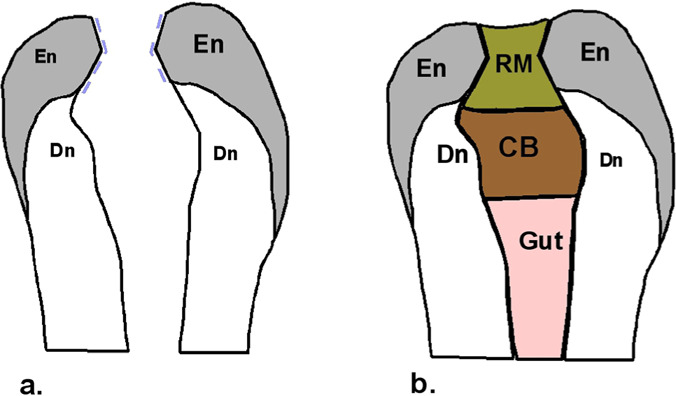
Sketch of an access cavity with an “hourglass” morphology, deliberately realized. a. The aspect of the access tunnel to the pulp chamber in the vertical section of the tooth. b. coronary and canal fillings of the tooth with such a preparation. En - enamel; Dn - dentin; RM - restorative material; CB - cement base; Gut - gutta-percha in the root canal.

The sketch of an access cavity with an “hourglass” morphology is presented in Figure 14.

## Conclusions

In order to verify the frequency of certain technical errors that occur in dental treatments and how significant these errors are, it would be necessary to study a large number of cases of extracted teeth to which endodontic and restorative treatment has been applied. Unfortunately, this is difficult and would only be possible with the participation of a large number of clinicians to provide such evidence for the study, as it is well known that not all patients are willing to donate extracted teeth for studies even though it is certain that identities will not be disclosed. The idea of presenting the clinical situation they experienced causes them a reluctance to give consent for the study. This is a common occurrence in our research practice.

Following the decision of longitudinal sectioning of the root to assess the quality of the canal filling, whole or part of the filling can be detached without the consent of the microtome operators. We appreciated the quality of the fusion of the gutta-percha cones used.

We concluded, without having data from clinical records, that the method used in root canal filling was warm lateral condensation, considering the good fusion of the cones in the center of the filling. However, the introduction of heated spreaders only in the central part of the bunch of cones makes it possible to clearly detect the boundaries between these cones towards the outside of the filling.

The vertical coronary enamel fissure, located in the middle of the vestibular aspect highlighted, cannot be interpreted as a result of the action of the wire hook of the mobilizable mandibular prosthesis worn by the patient, but as a consequence of the antagonist occlusal force relationship, also demonstrated by the wear lesion highlighted.

The nature of the coronary obstruction remains a question mark due to the high frequency of the air bubbles contained. Their presence suggests that the material used for access cavity closure is not a composite but rather a cement that has been spatulated for activation (probably a resin-modified glass ionomer cement).

The relationship of the coronary obturation with the occlusal enamel at its edge is well made towards the buccal and incorrectly made towards the lingual aspect. The polarized light used in association with the analyzer polarizing filter allowed us to detect these relationships in good conditions. The covering of the lingual cusp by a limbus of filling material is not correct through the step left with the enamel surface, although the passage seems to be smooth. Obviously, such a step is challenging to detect in clinical practice even with operating magnifiers, but it can be an element that later favors the filling fracture.

It is quite clear that the walls of the access cavity in the section plane are not easily convergent towards the apical, which suggests rather a slight hourglass shape.

## Conflict of Interest

The authors declare that there is no conflict of interest.
